# Supplementary material for: FNBP1 Facilitates Cervical Cancer Cell Survival by the Constitutive Activation of FAK/PI3K/AKT/mTOR Signaling
Source: Cells. 2023 Jul 29;12(15):1964. doi: 10.3390/cells12151964 (PMC10417648; doi:10.3390/cells12151964)
Supplement: Supplementary file 1 [file cells-12-01964-s001.zip › Caption Fig S1-S3.pdf]

Captions

Fig. S1 Construct and identification of FNBP1 shRNA vector. A. pGenesil-1 plasmid. B. Restriction map of shRNA vectors. M: DNA marker; 1: pGenesil-1; 2: Si-1; 3: Si-1digested with SalI; 4: Si-2; 5: Si-2 digested with SalI; 6: Si-3; 7: Si-3 digested with SalI; 8: NC; 9: NC digested with SalI. C. The partial sequence of recombinant shRNA plasmid. a: recombinant plasmid Si-1; b: recombinant plasmid Si-2; c: recombinant plasmid Si-3.

Fig.S2 Quantification of western blots in the manuscript. A. Quantification of the blots in Fig.1A and Fig.2A. B. Quantification of the blots in Fig. 2A. C. Quantification of the blots in Fig. 2B. D. Quantification of the blots in Fig. 3A. E. Quantification of the blots in Fig. 4A. F. Quantification of the blots in Fig. 5A

Fig. S3 Recover from FNBP1 silence. The relative quantification of FNBP1 expression, cell adhesion and contact area was determined as the following equation: Relative quantification (%) = FNBP1si/FNBP1control × 100%. The red group represented the relative expression of FNBP1 mRNA, the green one represented the relative adhesion capability of HeLa cells and the yellow one represented the relative cell contact area.
