# Supplementary material for: FNBP1 Facilitates Cervical Cancer Cell Survival by the Constitutive Activation of FAK/PI3K/AKT/mTOR Signaling
Source: Cells. 2023 Jul 29;12(15):1964. doi: 10.3390/cells12151964 (PMC10417648; doi:10.3390/cells12151964)
Supplement: Supplementary file 1 [file cells-12-01964-s001.zip › Tab.S1.pdf]

Tab. S1 Nucleotide sequences of shRNAs targeting FNBP1

shRNA	Sequences	Location NM_001363755.1
1	5'-gatcGCAGAAAGTCGATGAGTTAttcaagagaTAACTCATCGACTTTCTGCttttttgtcgaca-3'<br>3'-gCGTCTTTCAGCTACTCAATaagttctctATTGAGTAGCTGAAAGACGaaaaaacagctgttcga-5'	1464-1482
2	5'-gatcCCCACTTCATATGTCGAAGTCTGttcaagagaCAGACTTCGACATATGAAGTGGGttttttgtcgaca-3'<br>3'-gGGGTGAAGTATACAGCTTCAGACaagttctctGTCTGAAGCTGTATACTTCACCCaaaaaacagctgttcga-5'	2023-2045
3	5'-gatcCGGAGAAACATTGTATGTCAttcaagagaTGACATACAATGTTTCTCCttttttgtcgaca-3'<br>3'-gCCTCTTTGTAACATACAGTaagttctctACTGTATGTTACAAAGAGGaaaaaacagctgttcga-5'	1939-1957
