# Supplementary figures and images for: FNBP1 Facilitates Cervical Cancer Cell Survival by the Constitutive Activation of FAK/PI3K/AKT/mTOR Signaling
Source: Cells. 2023 Jul 29;12(15):1964. doi: 10.3390/cells12151964 (PMC10417648; doi:10.3390/cells12151964)

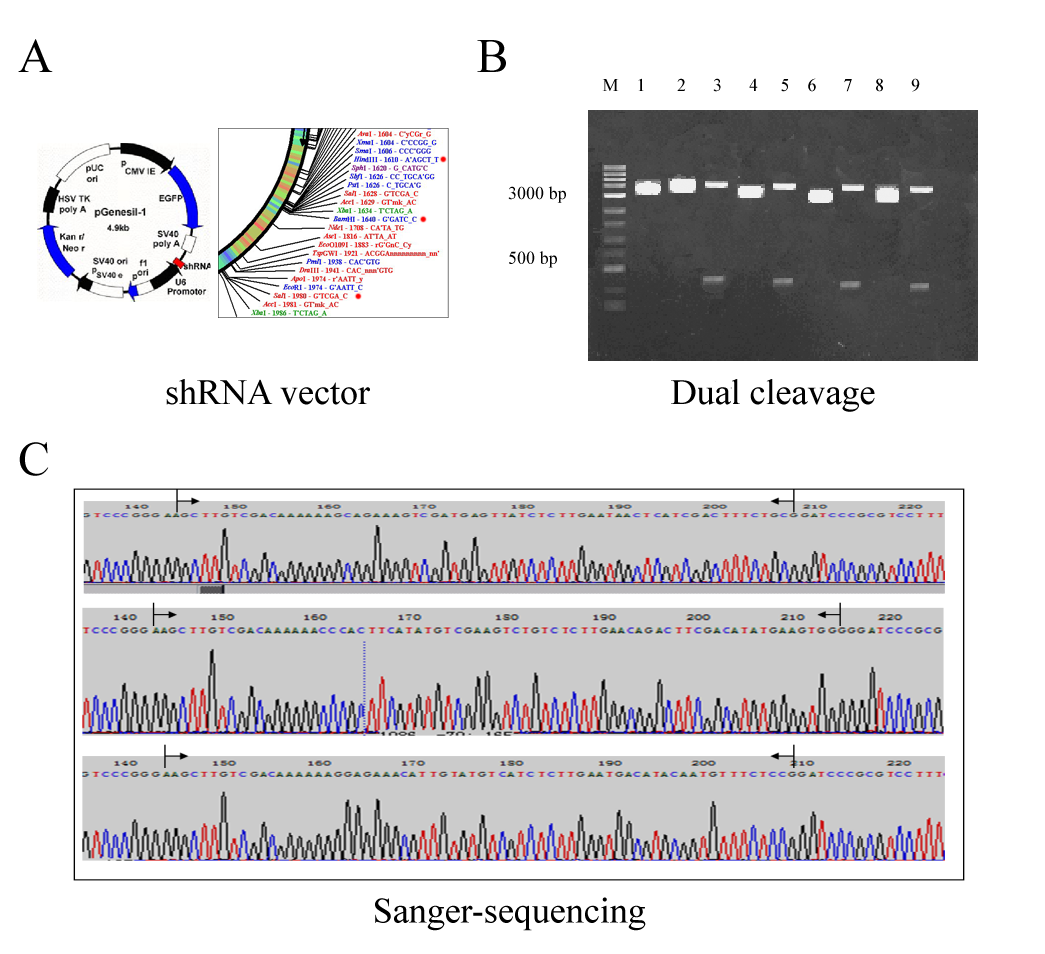

Supplement: Supplementary file 1 [file cells-12-01964-s001.zip › Fig S1-R.tif]

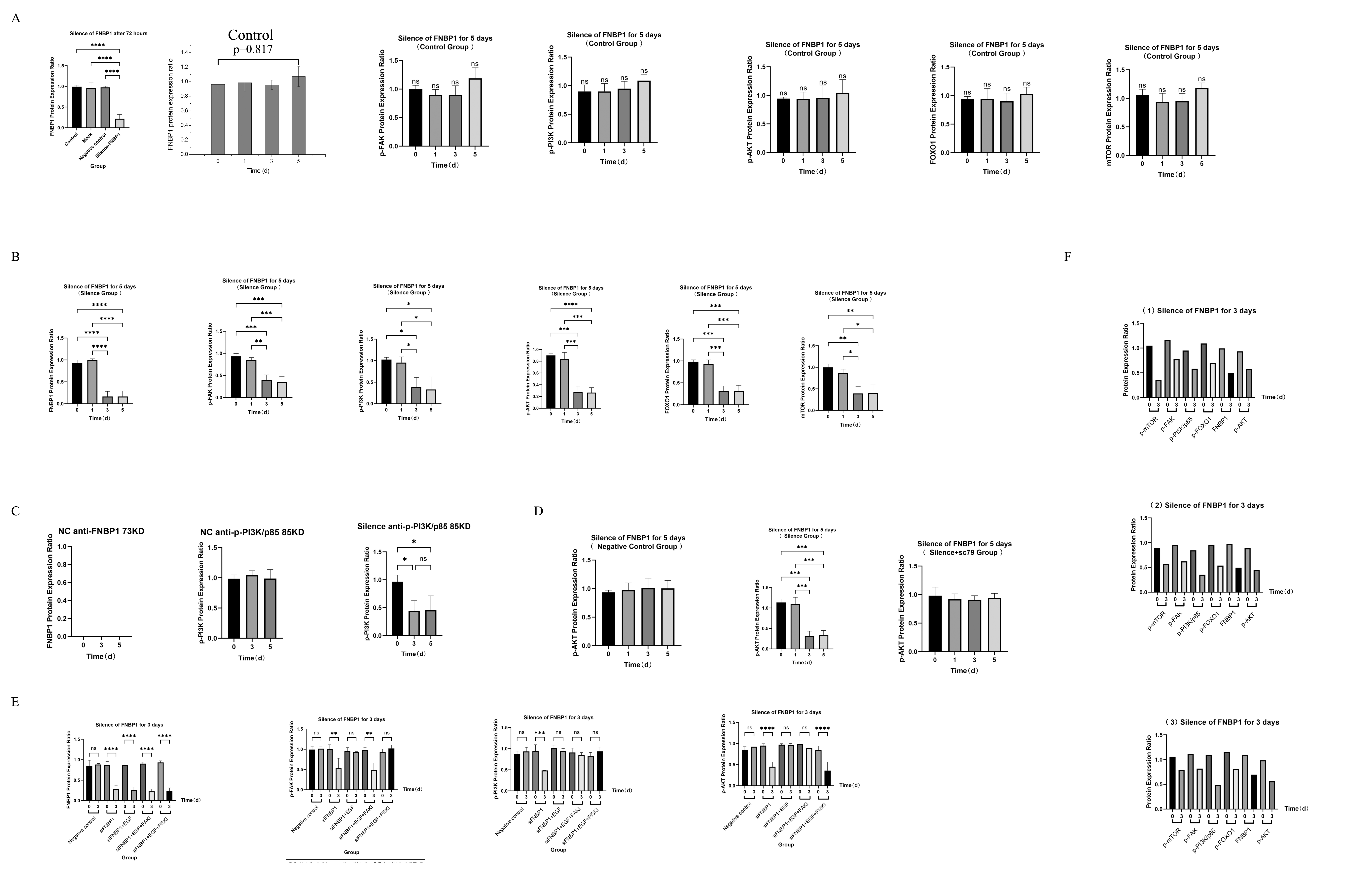

Supplement: Supplementary file 1 [file cells-12-01964-s001.zip › Fig. S2.tif]
